# Population-level insights into temporal interference for focused deep brain neuromodulation

**DOI:** 10.3389/fnhum.2024.1308549

**Published:** 2024-04-19

**Authors:** Kanata Yatsuda, Wenwei Yu, Jose Gomez-Tames

**Affiliations:** ^1^Department of Medical Engineering, Graduate School of Engineering, Chiba University, Chiba, Japan; ^2^Center for Frontier Medical Engineering, Chiba University, Chiba, Japan

**Keywords:** transcranial temporal interference stimulation, brain stimulation, group-level, electric field, deep brain, transcranial alternating current stimulation

## Abstract

The ability to stimulate deep brain regions in a focal manner brings new opportunities for treating brain disorders. Temporal interference (TI) stimulation has been suggested as a method to achieve focused stimulation in deep brain targets. Individual-level knowledge of the interferential currents has permitted personalizing TI montage via subject-specific digital human head models, facilitating the estimation of interferential electric currents in the brain. While this individual approach offers a high degree of personalization, the significant intra-and inter-individual variability among specific head models poses challenges when comparing electric-field doses. Furthermore, MRI acquisition to develop a personalized head model, followed by precise methods for placing the optimized electrode positions, is complex and not always available in various clinical settings. Instead, the registration of individual electric fields into brain templates has offered insights into population-level effects and enabled montage optimization using common scalp landmarks. However, population-level knowledge of the interferential currents remains scarce. This work aimed to investigate the effectiveness of targeting deep brain areas using TI in different populations. The results showed a trade-off between deep stimulation and unwanted cortical neuromodulation, which is target-dependent at the group level. A consistent modulated electric field appeared in the deep brain target when the same montage was applied in different populations. However, the performance in terms of focality and variability varied when the same montage was used among populations. Also, group-level TI exhibited greater focality than tACS, reducing unwanted neuromodulation volume in the cortical part by at least 1.5 times, albeit with higher variability. These results provide valuable population-level insights when considering TI montage selection.

## 1 Introduction

Non-invasive brain stimulation (NIBS) has generally been used to target superficial brain areas, but there has been increasing interest in noninvasive neuromodulation of deep focal brain parts that are strongly implicated in neurological and psychiatric disorders (Holtzheimer et al., 2017; Gunalan et al., 2018). Transcranial electrical stimulation (tES) and transcranial magnetic stimulation (TMS) are two conventional NIBS that can generate a significant electric current in deep brain areas (DaSilva et al., 2015; Csifcsák et al., 2018; Bikson and Dmochowski, 2020; Gomez-Tames et al., 2020a,b). However, the electric current falls off in both intensity and focality with increasing depth (Dmochowski et al., 2011; Deng et al., 2013).

**Figure 1:**
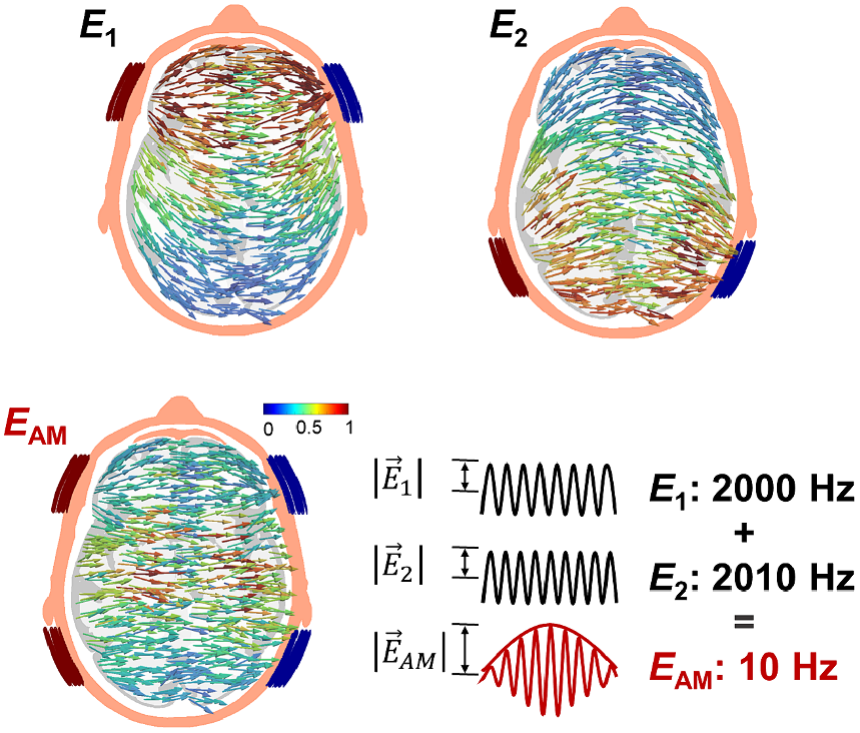
Transcranial temporal interference stimulation (TI) injects two-kilohertz currents of slightly different frequency via two pair of electrodes (*I*_1_ and *I*_2_) that produces an amplitude-modulated field pattern ${\vec{E}}_{\text{AM}}$.

Temporal interference (TI) stimulation has emerged as a promising alternative for focal deep stimulation while reducing undesired stimulation in nontarget areas. This is accomplished by superposing two-kilohertz alternating currents with slightly different frequencies (Grossman et al., 2017). In principle, the interferential region created by these two alternating currents (i.e., an amplitude modulation pattern with a low-frequency envelope) is considered to produce low-frequency neurostimulation, as illustrated in Figure 1. This phenomenon is attributed to the low-pass filtering properties of the neuron membrane accompanied by rectification of the ionic part (Grossman et al., 2017; Song et al., 2020). Previous studies have demonstrated a motor response corresponding to the interferential hotspot stimulus in the brain motor area of the mice (Grossman et al., 2017; Zhu et al., 2019; Song et al., 2020). In the case of humans, achieving such stimulation is prohibitive due to the substantial injection currents required, especially when considering the size differences between humans and mice (Alekseichuk et al., 2019; Rampersad et al., 2019). Instead, 5 Hz modulated TI has been found to have neuromodulatory effects on deep brain structures in humans (Li et al., 2019) resembling the effects of transcranial alternating current stimulation (tACS), a modality of tES that entrains ongoing brain oscillations and synchronizes neural networks.

Based on digital human head models, computational models have proven to be effective in predicting the *in-vivo* tES-generated electric field (or electric current) according to the anatomical/electrical properties of the tissues together with the selected montage configuration (Huang et al., 2017; Opitz et al., 2018; Jiang et al., 2022). Computational models have also revealed a significant relationship between the electric field and stimulation outcomes (Laakso et al., 2019; Hunold et al., 2023). Recently, computational analysis has been used to investigate the TI-generated electric field to optimize electrode locations (Huang and Parra, 2019; Rampersad et al., 2019), guide montage selection on humans (Li et al., 2019), quantify the differences with traditional tES montages (Rampersad et al., 2019), and clarify the mechanism of stimulation by including neural models (Mirzakhalili et al., 2020; Esmaeilpour et al., 2021; Gomez-Tames et al., 2021). This has led to individual-level knowledge of how the TI-generated electric field is shaped by the brain and non-brain variable structure and size (Rampersad et al., 2019; Lee et al., 2020) has been gained. While an individualized approach enables a high degree of personalization, the significant intra-and inter-individual variability among specific head models makes the comparison of electric-field doses difficult. Furthermore, the practical feasibility of acquiring MRI imaging for each individual to develop a personalized head model, followed by using navigation systems to localize optimized electrode positions, is not always available in various clinical settings, and it is restrictive in telemedicine applications where there is a need for simple and consistent electrode placement (Charvet et al., 2018).

The registration of individual electric fields into brain templates has offered insights into electric field characteristics at a population level (Fonov et al., 2011; Fischl, 2012). For example, understanding electric field dose variations between different populations (e.g., young vs. elderly) allows us to tailor stimulation parameters to the specific needs of the target population (population-level knowledge of the electric field). Also, this facilitates the determination of the best common montage for all participants based on common scalp landmarks (international 10–10 system positioning) (Laakso et al., 2016; Gomez-Tames et al., 2019; Rezaee and Dutta, 2019; Gomez-Tames et al., 2020a; Soleimani et al., 2021). Population-level knowledge of the electric field eliminates the need for MRI acquisition and localization based on a navigation system. Additionally, it avoids extending participants’ time in the experiment, aligning with current trends in telemedicine. However, in the case of TI, it remains largely unexplored how effectively TI can consistently target small deep human brain structures compared to other conventional montages, except in a single study (von Conta et al., 2021). Moreover, the application of population-level TI across different demographic groups has yet to be explored, given the potential application of TI, including improving cognitive functions in the elderly population. Also, populations with affected brain areas need to be investigated for TI. This becomes relevant considering the potential differences in the group-level electric field between the healthy and some clinical populations in tES (Mizutani-Tiebel et al., 2022). Stroke patients, a target population for alternating current stimulation (Naros and Gharabaghi, 2017; Fresnoza et al., 2018; Khan et al., 2022), present significant intracranial structure changes that may affect the electric current distributions during TES (Minjoli et al., 2017; Carla Piastra et al., 2021), yet this aspect remains to be explored for TI.

This work investigated the effectivity of targeting deep brain areas using TI modulation. For that, we conducted electric field analyses to explore focality and investigate whether the montage could consistently stimulate deep brain structures at the population level. We used a high-resolution computational model with registration techniques in neurotypical and non-neurotypical brains.

## 2 Model and methods

### 2.1 Head models

**Figure 2:**
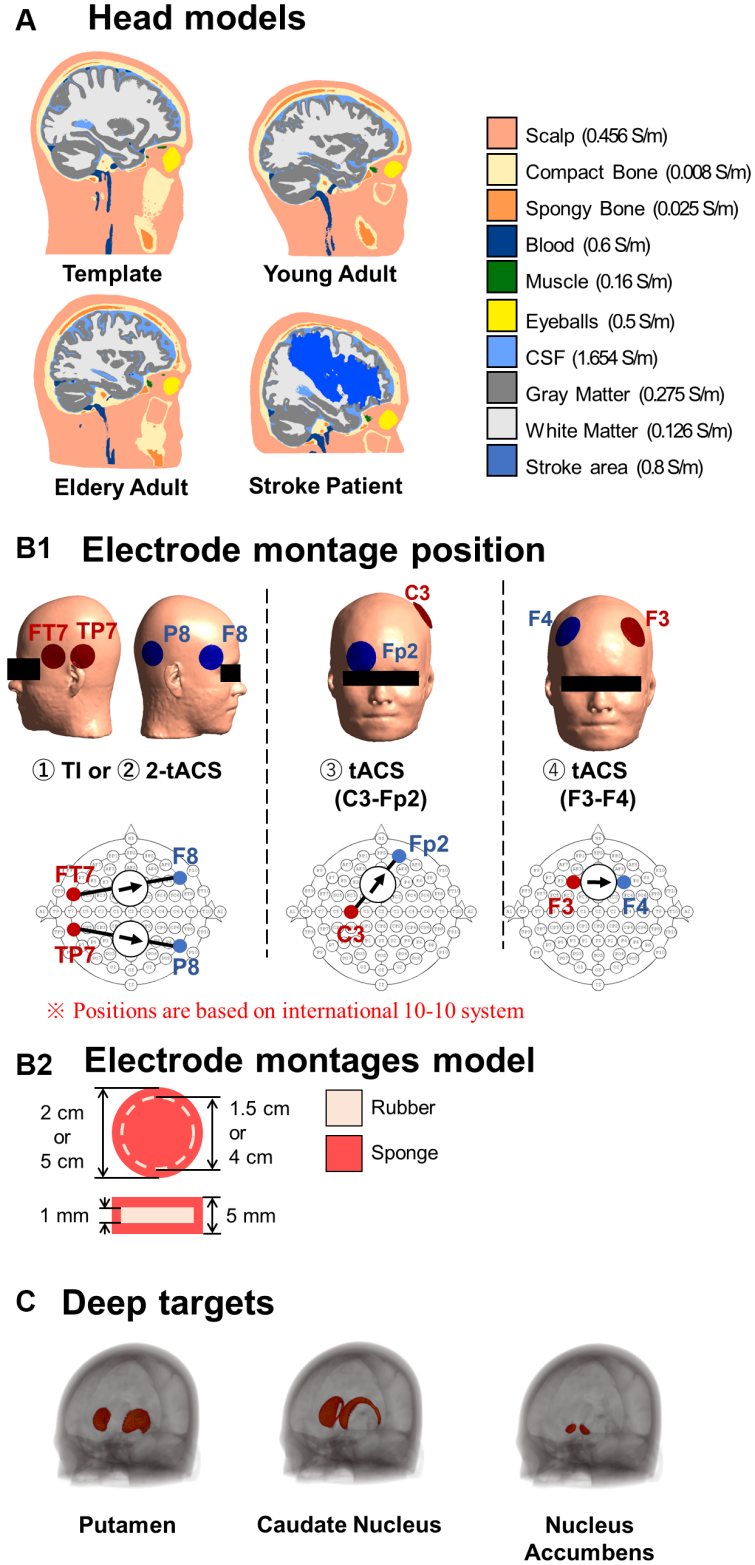
**(A)** Standard head models and representative head models of different populations. **(B1)** The electrode montages were placed according to the international 10–10 system, based on for the two stimulations schemes. Temporal interference (TI) electrode montages utilize two pairs of electrodes to inject and produce a modulated current in the brain. Transcranial alternating current stimulation (tACS) montages generate a non-modulated current using either two pairs of electrodes (2-tACS) or one pair of electrodes (tACS). **(B2)** All electrodes are constructed with rubber and sponge materials. **(C)** Deep brain regions of interest.

A template head model was employed to investigate electric fields generated in its brain template (MNI152 from 152 normal adults, 18.5–43.5 years). Also, the head models of 120 individuals were constructed from magnetic resonance images (MRIs) using SIMNIBS/charm to investigate the electric field at the individual level (Puonti et al., 2020). The data was obtained from freely available repositories, as shown in Table 1. The MRI data of each individual were segmented into ten tissues, including non-brain, brain, and fluids, as illustrated in Figure 2A. In the case of stroke patients (Liew et al., 2018), manually obtained lesion masks were added to the segmented head model. The electrical conductivities of the scalp, compact bone, spongy bone, blood, muscle, eyeballs CSF, gray matter, and white matter were 0.456, 0.008, 0.025, 0.6, 0.16, 0.5, 1.654, 0.275, 0.126 S/m, respectively (Gonçalves et al., 2003; Wagner et al., 2004; Dannhauer et al., 2011). The conductivity of the stroke area was 0.8 S/m (Jiang et al., 2022). The conductivities represent average values used in similar studies (Rampersad et al., 2019; von Conta et al., 2021).

**Table 1:** Head models generated for three populations based on MRI T1 and T2 data.

Head model	Population
Brain template (MNI152) (Fonov et al., 2011)	Averaged from normative young adults
Neurotypical young adult^1^	20 males and 20 females (23.0 ± 2.9 years)
Neurotypical elder adult^1^	20 males and 20 females (70.0 ± 3.2 years)
Non-neurotypical adult^2^	40 stroke patients

^1^https://openneuro.org/; ^2^https://fcon_1000.projects.nitrc.org/indi/retro/atlas.html.

### 2.2 Electrode montages

The electrode montages are shown in Figure 2B. The electrode model consisted of a 1-mm-thick rubber sheet (conductivity of 0.1 S/m) within a sponge soaked in normal saline solution (1.6 S/m) (Dundas et al., 2007; Saturnino et al., 2015) for a total thickness of 5 mm. The current source or sink was placed in the rubber. The TI montage was two bipolar electrodes with an injection current of 1 mA (total injection current of 2 mA) and an area of 3.14 cm^2^. Initially, the electrode positions corresponded to the montage used for TI modulation for the human deep brain area (TP7-P8, FT7-F8) (Violante et al., 2023). The electrodes’ positions were according to the international 10–10 system, which is used as a standardized method for EEG electrode localization in the scalp. In addition, a total of 2,250 montages were investigated using two grids centered on TP7 and FT7 to determine variations in the delivered interferential current on different deep brain targets.

TI was compared with three tACS montages. Two of them were bipolar montages located at positions that can systematically generate significant hotspots in deep brain regions in a population (C3-Fp2 and F3-F4) (Gomez-Tames et al., 2020a; Hamajima et al., 2023). The third tACS montage consisted of two bipolar montages without phase difference and placed in identical locations as the TI montage, as a control montage for TI. The total current injected current was 2 mA for any montage. The area of the electrodes was the same for all montages (19.63 cm^2^) to keep the same current density and reach deep areas with significant intensities (Fresnoza et al., 2018; Kasten et al., 2019; Gomez-Tames et al., 2020a).

### 2.3 Deep brain targets

Three deep brain areas were investigated. The putamen is a round structure (3.56 ± 0.10 cm^2^) that is critical for the execution of motor behavior (Middleton and Strick, 2000). The caudate nucleus has a C-shape form (3.00 ± 0.15 cm^2^) involved in various goal-directed behavior and cognitive functions and has similar associated disorders (Brown et al., 2004). The nucleus accumbens is a small round structure (1.72 ± 0.10 cm^2^) that is a key part of the reward system (Volkow et al., 2017). The different sizes and shapes of these different functional deep structures (Gomez-Tames et al., 2020b) served as representative factors for evaluating the performance of TI. These deep brain structures were obtained from an available developed deep brain atlas in brain template (MNI-152) (Pauli et al., 2018).

### 2.4 Electric field model

The electric field ($\vec{E}$) generated by the current injected from a pair of electrodes attached to the scalp was computed using the finite element method (SimNIBS 4.0) (Thielscher et al., 2015) considering quasi-static approximation (Plonsey and Heppner, 1967) to solve the Laplace equation:


(1) $$\nabla .\left(\sigma \nabla \varphi \right)=0,$$

where φ and σ denote the scalar potential and tissue conductivity, respectively. The electric field $\vec{E}=-\nabla \varphi$ was calculated in each element.

The same conductivity was used for tACS and TI (listed in 2.1), considering similar values for their operation frequency (Brown et al., 2004). The top 0.01 percentile values were removed within the target tissue to remove potential electric field outliers (Gomez-Tames et al., 2017; Soldati and Laakso, 2020).

The volume conductor was divided into spatial elements and nodes for discrete computations. The tetrahedral head meshes for FEM were generated using the ‘mri2mesh’ tool in the SimNIBS software pipeline with an average of 3.5 million facets per head with an average edge length of 2.1 mm. Dirichlet boundary conditions on electrode surfaces and homogeneous Neumann boundary conditions elsewhere are provided. SimNIBS solves the linear system using an iterative preconditioned conjugate gradient method (Hunold et al., 2023).

A bipolar tACS-generated electric field distribution is obtained using Equation 1. The electric field of two simultaneous tACS bipolar electrodes (referred to as 2-tACS) is obtained by the vector sum for the in-phase condition. Additional calculation steps were necessary to examine the distribution of the total electric field generated from the temporal interfering fields of two electrode pairs. The spatial distribution of the amplitude-modulated envelope (*E*_AM_) is obtained by Equation 2, which is derived from the vector sum of the two resulting electric fields (*E*_1_ and *E*_2_) with a phase difference (Grossman et al., 2017; Rampersad et al., 2019; von Conta et al., 2021).


(2) $$\begin{array}{l} |{\vec{E}}_{\text{AM}}\left(\vec{r}\right)|=\\ \left\{ \begin{array}{l} 2|{\vec{E}}_{2}\left(\vec{r}\right)|\;\text{if}\;|{\vec{E}}_{2}\left(\vec{r}\right)|<|{\vec{E}}_{1}\left(\vec{r}\right)|\text{cos}\alpha \\ 2|{\vec{E}}_{2}\left(\vec{r}\right)\times \left({\vec{E}}_{1}\left(\vec{r}\right)-{\vec{E}}_{2}\left(\vec{r}\right)\right)/|{\vec{E}}_{1}\left(\vec{r}\right)-{\vec{E}}_{2}\left(\vec{r}\right)||\;\text{otherwise}\; \end{array}\right. \end{array}$$

where ${\vec{E}}_{1}\left(\vec{r}\right)$ and ${\vec{E}}_{2}\left(\vec{r}\right)$ correspond to the electric fields at position $\vec{r}$. If $\left|\vec{E_{1}}\right|$ > $\left|\vec{E_{2}}\right|$ and the angle (angle between $\left|\vec{E_{1}}\right|$ and $\left|\vec{E_{2}}\right|$) is smaller than 90°, the maximal modulation amplitude is obtained. If the angle is more than 90°, the sign must be reversed. This is because the resulting maximum TI field strength in the brain is a combination of two time points in one oscillation, as implemented in the SimNIBS toolbox (von Conta et al., 2021). The electric field calculation after interference for a given montage took approximately 5 min on an HP ProDesk 600 G6 Small Form Factor PC.

### 2.5 Analysis methods

**Figure 3:**
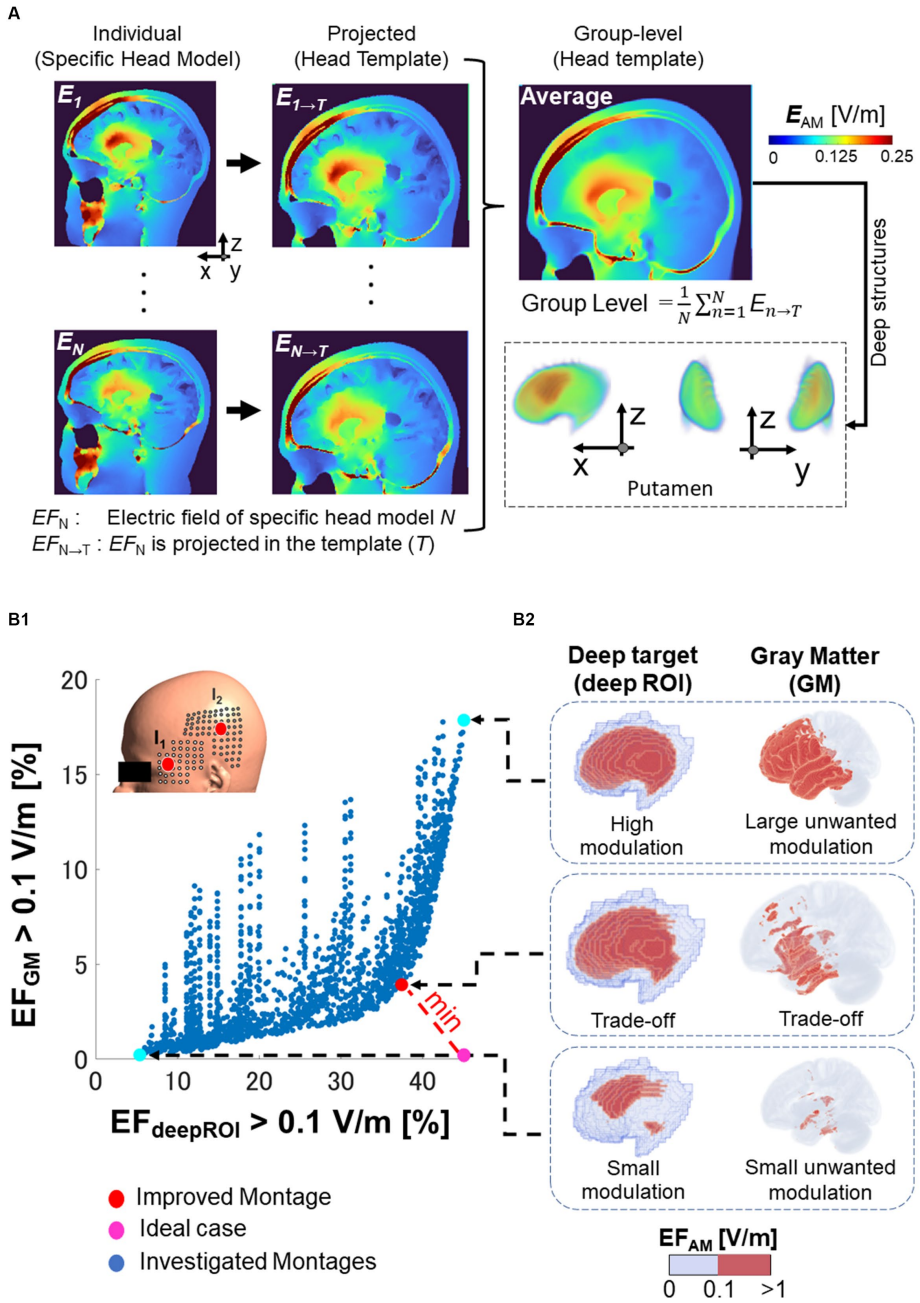
Electric field analysis. **(A)** Normalization of the electric field (EF) of specific brain anatomical model of the subject *N* (*EF*_N_) to a common brain template (*EF*_N → T_). The group level is the resulting average of the electric fields normalized to the template. **(B1)** For various montages on the grid (each blue dot), a trade-off appears between neuromodulation volume percentage in the deep brain target (deepROI) and neuromodulation volume percentage in the gray matter (GM), as shown in the x-and y-axis, respectively. The selected improved montage is the one with the shortest distance from the ideal case (i.e., a maximum field in deepROI and minimum field in GM). **(B2)** The neuromodulation volumes are illustrated in the right side for the putamen as one deep target (wanted modulation volume) and in the GM (unwanted neuromodulation volume).

The neuromodulation quantity was the modulated electric field $\left|{\vec{E}}_{\text{AM}}\right|$ in the case of TI or peak electric field (*E*) for unmodulated stimulation (i.e., tACS) (Antal and Herrmann, 2016; Grossman et al., 2017) for each individual. The substantial intra-inter-variability hinders spatial comparison, requiring methods for determining population-level knowledge of electric field distributions. Initially, individual electric fields were estimated in the specific head model (individual head models from young, elderly, or stroke patients) and normalized to the head template (MNI152 space) using the subject2mni function from SimNIBS, as shown in Figure 3A. Normalization to the head template makes it possible to calculate the average electric field across multiple subjects (group-level effect in a population). Analyzing group-level electric fields provides insights into the population-level knowledge of electric field distributions.

To investigate focality in the target region, we estimate the volume where the electric field exceeds a neuromodulation threshold (called neuromodulation volume: $EF_{ROI}>\text{Threshold}\;\left[V/m\right]$). The neuromodulation volume was obtained in the following regions of interest (ROI): gray matter (GM) and the three deep brain areas on the left hemisphere (deepROI). A trade-off is formed between the neuromodulation volumes in the gray matter and the deep target, as illustrated in Figure 3B. The ideal case was set as the one in which both neuromodulation volumes would result in an optimal outcome. The electrode configuration closest to the ideal case in trade-off was chosen as the improved montage. In other words, the best focality corresponds to the stimulation that maximizes the neuromodulation volume on the deep brain region and minimizes the neuromodulation volume on the gray matter (unwanted neuromodulation). This process can be used to ascertain the optimal electrode configuration to determine highly focal placements for each deep brain target.

For the neuromodulation threshold, a minimum threshold of 0.1 V/m has been reported to generate tACS modulation effects based on intracranial measurements and individualized electric field modeling in monkeys [0.12 V/m (Kar et al., 2017)] and human [0.13 V/m, 0.08 to 0.36 V/m (Puonti et al., 2020)] which are within the range that modulates neural activity *in vitro* and *in vivo* studies (Francis et al., 2003; Ozen et al., 2010; Fonov et al., 2011).

## 3 Results

### 3.1 Group-level effect of TI on deep structures

**Figure 4:**
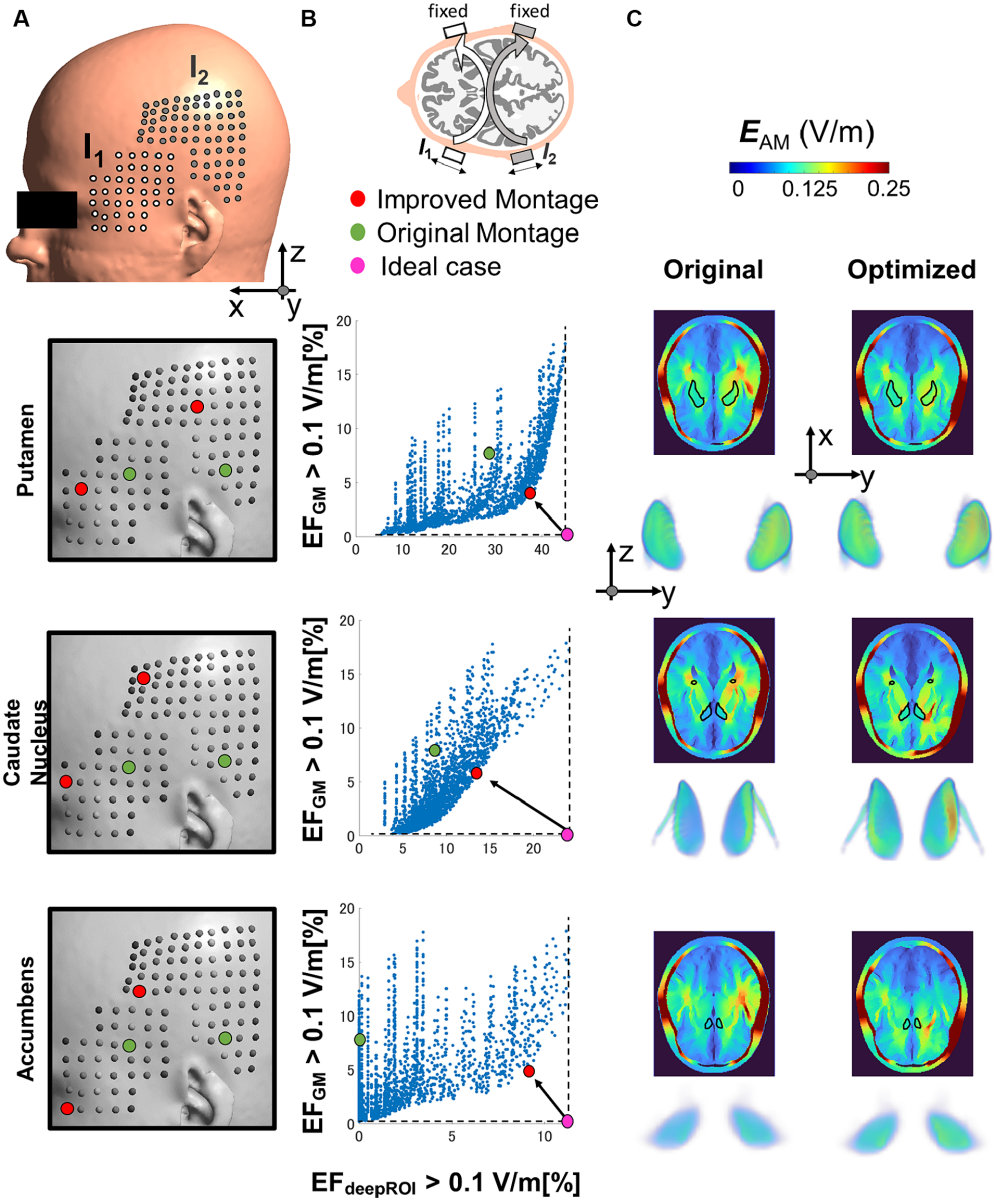
**(A)** The optimal electrode location is determined by considering various electrode positions within the grid on the temporal/parietal side. The grid is centered on the initial montage position based on (Violante et al., 2023). **(B)** The optimal montage is selected by minimizing the percentage of neuromodulation volumes in the gray matter (GM) and maximizing the percentage of neuromodulation volume in the deep region of interest (deepROI). **(C)** Electric field (EF) distributions are depicted for both the selected and original montage configurations across three deep areas (putamen, caudate nucleus, and accumbens).

Figure 4A shows the deep focal neuromodulation performance of TI montage using the head template model. It shows the neuromodulation volume for gray matter and each deep brain region for a total of 2,250 montages based on the combination of electrodes located on a grid centered on a reference TI montage (Figure 4A). Figure 4B shows the focality for the different montage combinations, as the relationship between the neuromodulation volumes (*E*_AM(GM)_ > 0.1 V/m) and deep brain region (*E*_AM(deepROI)_ > 0.1 V/m). A trade-off between both neuromodulation volumes is presented, forming a Pareto front (Figure 4B).

We select the best montage using the minimum distance between the Pareto front and maximum focality. Figures 4B,C show that the improved montage location differs according to the deep brain target despite their relatively close location. The neuromodulation volume in the gray matter (unwanted stimulation) is reduced by a percentage change between 40 and 50% for the three deep targets from the reference. Also, the neuromodulation volume in the deep brain areas increased by a percentage change between 30 to 800%. For the latter, the improvements were significantly important for the small deep brain target (nucleus accumbens). It is also possible to make the selection by a different criterion, such as minimizing the neuromodulation volume in the gray matter while keeping the same neuromodulation volume in the deep brain target (reduction by four and eight times for the putamen and caudate nucleus, respectively).

### 3.2 Dependency on target population

**Figure 5:**
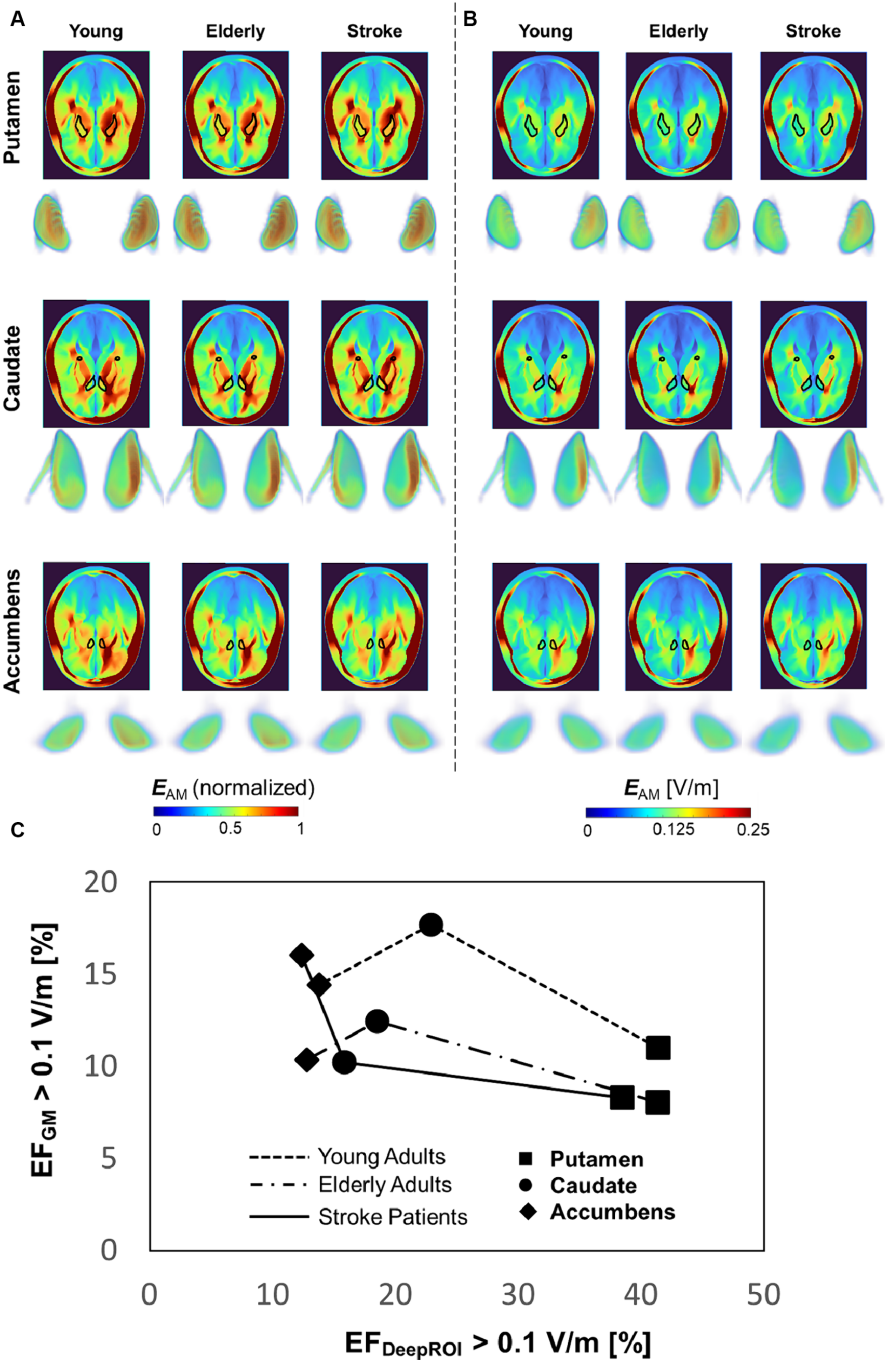
**(A,B)** Group-level comparison between populations for normalized and non-normalized electric field in the head (cross-section, superior view) and deep brain targets (3D data, frontal view). **(C)** Focality graph showing the relationship between neuromodulation volumes in the gray matter and targets for different populations.

Figure 5A shows the group-level modulated electric field of young, elderly, and stroke populations for TI. The electric fields are shown in a cross-section of the head, along with the 3D data of three deep brain targets for both non-normalize and normalized data. The group-level electric field exhibits disguisable hotspots in the normalized data hotspot distributions of the three deep targets of different populations. The non-normalized data shows that the fields in the accumbens are weaker than in the other two deep brain areas. Figure 5B shows a closer look into neuromodulation volume at each deep brain target. The results show that the neuromodulation volume in gray matter is higher in the young population than in the elderly and stroke populations when targeting the caudate and putamen. Additionally, the neuromodulation volume is slightly higher in the young population in caudate and accumbens targets. Regarding variability of the neuromodulation volumes, Table 2 shows marginal differences between young and elderly populations for deep brain targets. There was higher variability in the stroke group, as expected.

**Table 2:** Relative standard deviation (RSD) of neuromodulation volumes in different populations using TI montage.

	RSD [volume (EF_ROI_ > 0.1 V/m)] [%]		
ROI	Young	Elderly	Stroke
Putamen	12.8	12.6	20.4
Caudate	28.8	26.0	33.8
Accumbens	25.5	27.5	20.8
GM^*^	40.3–54.4	52.6–67.6	22.8–40.2

^*^A range is reported for GM considering each deep brain target. TI and 2-tACS used the same montage.

### 3.3 Population-level montages comparison

**Figure 6:**
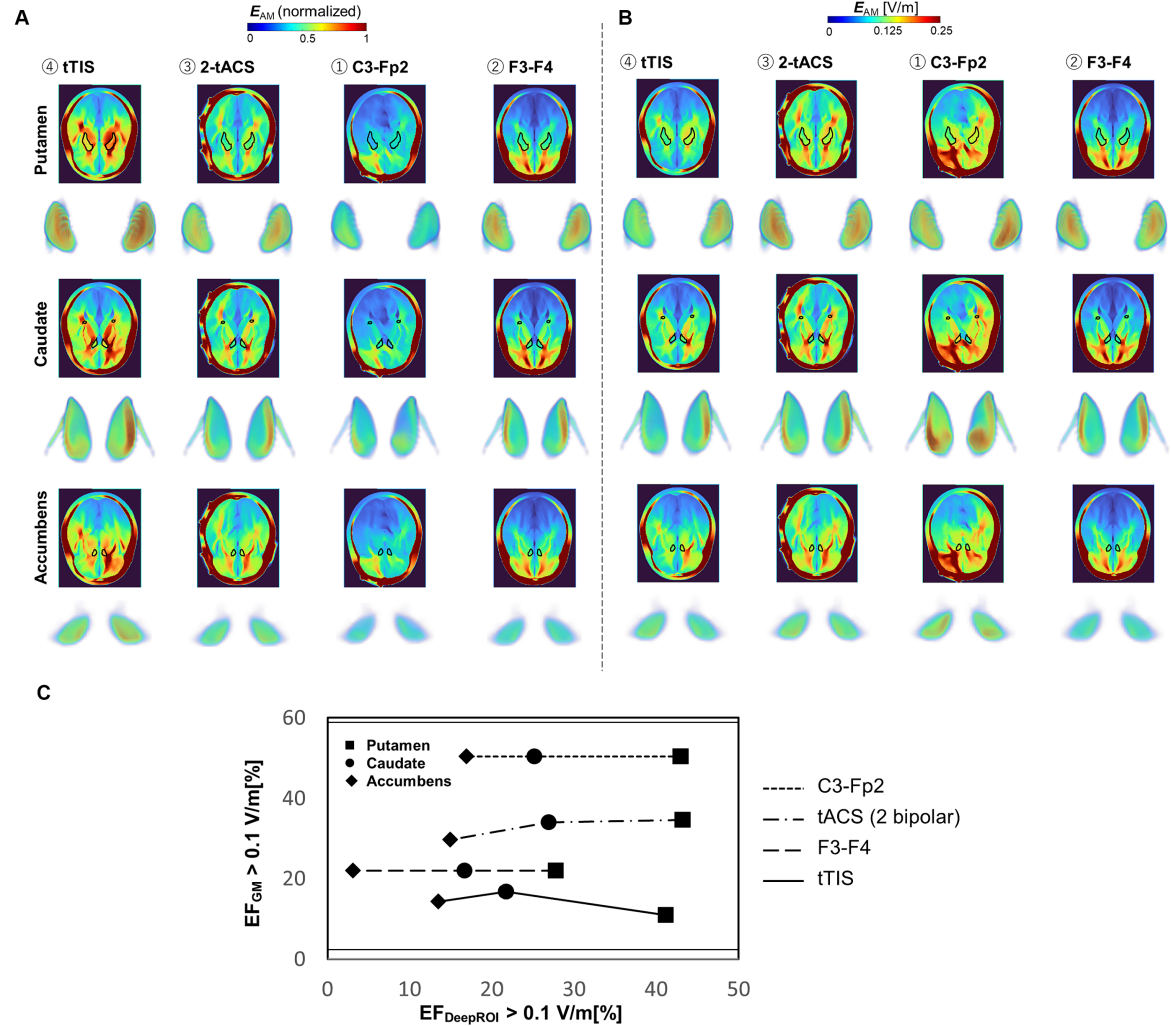
**(A,B)** Group-level electric field distribution comparison between different montages (*n* = 40, young population) for normalized and non-normalized electric fields. **(C)** Focality graph showing the relationship between neuromodulation volumes in the gray matter and targets for different montages.

Figure 6A shows the group-level electric field for TI and tACS montages in a young adult population. TI is shown to be more effective in generating clear hotspots in deep brain parts than two classic bipolar montages (C3-Fp2 and F3-F4) and control (2-tACS) for normalized data. TI also presents advantages for generating focalized fields in deep targets, although weaker fields are presented compared to the other montages observed from normalized data. Figure 6B shows that TI can reduce the neuromodulation volume in the gray matter by at least 1.5 times compared to 2-tACS or at least 1.3 times compared with the two bipolar montages.

Regarding the variability of the neuromodulation volumes, Table 3 shows a generally larger neuromodulation variability in the TI montage than in the tACS montages in gray matter and deep brain targets. The variability increased significantly in deep brain targets compared to gray matter for the different montages, particularly for TI. This shows the importance of controlling the TI dose more than the tACS montages.

**Table 3:** Relative standard deviation of neuromodulation volumes using different montages.

	RSD [volume (EF_ROI_ > 0.1 V/m)] [%]			
ROI	TI	2-tACS	C3-Fp2	F3-F4
Putamen	13.1	7.3	5.3	26.3
Caudate	29.4	21.2	17.9	39.3
Accumbens	25.4	19.9	19.4	116.5
GM	38.9–53.5^*^	29.8–33.6^*^	15.7	24.8

^*^A range is reported for GM considering each deep brain target. TI and 2-tACS used the same montage.

## 4 Discussion

Invasive electrical deep brain stimulation (DBS) has been used to treat patients with severe movement disorders (Elias et al., 2018). However, the risks associated with brain surgery make exploring different brain targets difficult and limit potential therapeutic impact. The ability to non-invasively stimulate deep brain regions in a focal manner brings new opportunities for treating brain disorders (Bikson and Dmochowski, 2020). In this study, we investigated the population-level effectiveness of focal deep brain structures targeting via TI stimulation in different groups of subjects for the first time.

Focality was used to guide the selection of montage location on an averaged brain model (human head template model). Focality refers to maximizing the neuromodulation volume in deep brain areas and minimizing the neuromodulation volume on the gray matter. A trade-off between both neuromodulation volumes emerged and formed a Pareto-front for the different combinations of the electrode locations (Figure 4) in agreement with other studies (von Conta et al., 2021). The best electrode location varied between deep brain targets despite their relative proximity. Variability of the Pareto fronts has been found among deep brain targets in agreement with (Wang et al., 2023). The importance of montage selection becomes particularly evident when aiming for improved focality of small deep brain targets, such as the nucleus accumbens. It is worth noting that the modulation volume on the gray matter can be minimized to values near zero (fundamental for achieving true deep focal stimulation) at the expense of a smaller neuromodulation volume. We need to determine how much the TI injection current can be increased so the electric field reaches neuromodulation levels when the neuromodulation volume in gray matter is minimized.

Personalized TI at the individual level has been suggested to enhance its efficacy, considering the variations in the optimal montage location among a limited number of head models (Lee et al., 2020). However, given that several computational studies have shown significant and focal fields are produced in deep brain targets at the individual level for TI (Huang and Parra, 2019; Rampersad et al., 2019) and the feasibility of achieving consistent targeting with conventional (unmodulated) tES stimulation at the group level (Kasten et al., 2019), it is important to investigate TI at group-level. Surprisingly, population-level knowledge of interferential currents for TI remains largely unexplored, except for one study (von Conta et al., 2021). Moreover, the application of population-level TI across different demographic groups has yet to be explored (Naros and Gharabaghi, 2017). In this study, we applied the same montage location for all head models in the different populations based on the international 10–10 system localization. The results show that despite the individual differences, it was possible to have a consistent group-level modulated electric field in the deep brain targets (Figures 5, 6). This demonstrates that TI can be applied using “one-for-all,” which can be applied in various clinical settings. However, differences were found between populations in the group-level electric field distribution and focality. Also, there were differences in the variability of the neuromodulation volume variability. Stroke patients had higher variability of neuromodulation volume in deep brain targets than young and elderly populations due to the inherent inter-variability of the affected areas. The elderly population had a higher variability of neuromodulation volume in the gray matter. In addition, we compared group-level electric fields between TI and tACS montages. The TI montage had better focality than a 2-tACS, both with the same electrode position, as well as two classic bipolar montages. The focality difference between TI and 2-tACS primarily resulted from a smaller neuromodulation volume in the cortex (unwanted co-stimulation) rather than variations in deep brain targets, which was also observed by von Conta et al. (2021). On the other hand, TI had higher variability of the neuromodulation volume than tACS in the different ROIs.

The current study has various limitations. First, the study presented here is purely numerical and is based on head models with only ten tissues, which may oversimplify some structures. Second, modeling approaches typically assume tissue conductivity to be constant across individuals, as in the current study (Indahlastari et al., 2020; Antonenko et al., 2021). However, the effects of aging on conductivity may impact the estimated electric fields. One study found a reduction in intracranial electric field intensity due to a reduction in skull conductivity with age (McCann and Beltrachini, 2021). Thus, incorporating age-appropriate skull conductivity in the models may enhance the observed electric field magnitude reduction in the elderly population (due to the loss of grey matter volume), as shown in our study and others (Antonenko et al., 2021). In the case of CSF, its conductivity remains relatively stable (Baumann et al., 1997). Further conductivity data from intracranial tissues is needed to understand better how age conductivity changes affect electric field analysis to enable age-appropriate conductivities in future computational modeling. Third, the head model did not consider the anisotropic effects of the conductivity of brain tissues, in particular for the white matter, that could yield stronger hotspots in deep brain structures (Katoch et al., 2023). Despite these limitations, it is important to note that electric field analysis methods have been able to reproduce *in vivo* measurements of intracranial currents (Puonti et al., 2020; Wang et al., 2022). Nevertheless, we acknowledge the need for detailed experimental validation to enhance the accuracy of electric field computation and improve parameter fitting, particularly when considering aging effects and non-typical brains.

Finally, we explored montage location based on an initial reference montage used for effective TI modulation in the deep brain. Future studies may adopt optimization studies to investigate optimized focality considering all scalp positions (Fernández-Corazza et al., 2020; Stoupis and Samaras, 2022). Also, TI and 2-tACS used the same montage location for fair comparison. This comparison does not consider differences in the stimulation waveforms or the effect of high-frequency components on neuronal modulation and should be considered in future work (Mirzakhalili et al., 2020). In addition, future studies need to investigate the impact of the arrangement and orientation of neurons in deep fibers relative to the induced electric field in deep structures (Richter et al., 2013).

## 5 Conclusion

This study contributes innovative population-level insights for selecting Temporal Interference (TI) montage. Firstly, a consistently modulated electric field is observed at the group level in deep brain regions. Secondly, the study demonstrates that the group-level electric field generated by TI offers advantages over its tACS counterpart, particularly in reducing undesired stimulation in the gray matter. Finally, the research highlights the importance of group-level montage selection based on the target population and the specific deep brain target.

## Data availability statement

Publicly available datasets were analyzed in this study. This data can be found here: https://openneuro.org/, https://fcon_1000.projects.nitrc.org/indi/retro/atlas.html.

## Author contributions

KY: Data curation, Formal analysis, Investigation, Methodology, Writing – review & editing, Software, Visualization. WY: Data curation, Methodology, Writing – review & editing, Supervision. JG-T: Data curation, Methodology, Supervision, Writing – review & editing, Conceptualization, Formal analysis, Funding acquisition, Investigation, Resources, Writing – original draft.

## References

[ref1] Alekseichuk I. Mantell K. Shirinpour S. Opitz A. (2019). Comparative modeling of transcranial magnetic and electric stimulation in mouse, monkey, and human. NeuroImage 194, 136–148. doi: 10.1016/j.neuroimage.2019.03.044, PMID: 30910725 PMC6536349

[ref2] Antal A. Herrmann C. S. (2016). Transcranial alternating current and random noise stimulation: possible mechanisms. Neural Plast 2016, 1–12. doi: 10.1155/2016/3616807 PMC4868897 27242932

[ref3] Antonenko D. Grittner U. Saturnino G. Nierhaus T. Thielscher A. Flöel A. (2021). Inter-individual and age-dependent variability in simulated electric fields induced by conventional transcranial electrical stimulation. NeuroImage 224:117413. doi: 10.1016/j.neuroimage.2020.117413, PMID: 33011418

[ref4] Baumann S. B. Wozny D. R. Kelly S. K. Meno F. M. (1997). The electrical conductivity of human cerebrospinal fluid at body temperature. IEEE Trans Biomed Eng 44, 220–223. doi: 10.1109/10.554770 9216137

[ref5] Bikson M. Dmochowski J. (2020). What it means to go deep with non-invasive brain stimulation. Clin. Neurophysiol. 131, 752–754. doi: 10.1016/j.clinph.2019.12.003 31917081

[ref6] Brown J. W. Bullock D. Grossberg S. (2004). How laminar frontal cortex and basal ganglia circuits interact to control planned and reactive saccades. Neural Netw. 17, 471–510. doi: 10.1016/j.neunet.2003.08.006, PMID: 15109680

[ref7] Carla Piastra M. van der Cruijsen J. Piai V. Jeukens F. E. M. Manoochehri M. Schouten A. C.. (2021). ASH: an automatic pipeline to generate realistic and individualized chronic stroke volume conduction head models. JNE 18:044001. doi: 10.1088/1741-2552/abf00b 33735847

[ref8] Charvet L. Shaw M. Dobbs B. Frontario A. Sherman K. Bikson M.. (2018). Remotely supervised transcranial direct current stimulation increases the benefit of at-home cognitive training in multiple sclerosis. Neuromodulation 21, 383–389. doi: 10.1111/ner.12583, PMID: 28225155 PMC5975186

[ref9] Csifcsák G. Boayue N. M. Puonti O. Thielscher A. Mittner M. (2018). Effects of transcranial direct current stimulation for treating depression: a modeling study. J. Affect. Disord. 234, 164–173. doi: 10.1016/j.jad.2018.02.077 29529550

[ref10] Dannhauer M. Lanfer B. Wolters C. H. Knösche T. R. (2011). Modeling of the human skull in EEG source analysis. Hum Brain Mapp 32, 1383–1399. doi: 10.1002/hbm.21114, PMID: 20690140 PMC6869856

[ref11] DaSilva A. F. Truong D. Q. DosSantos M. F. Toback R. L. Datta A. Bikson M. (2015). State-of-art neuroanatomical target analysis of high-definition and conventional tDCS montages used for migraine and pain control. Front. Neuroanat. 9:89. doi: 10.3389/fnana.2015.00089 26236199 PMC4502355

[ref12] Deng Z. D. Lisanby S. H. Peterchev A. V. (2013). Electric field depth–focality tradeoff in transcranial magnetic stimulation: simulation comparison of 50 coil designs. Brain Stimul. 6, 1–13. doi: 10.1016/j.brs.2012.02.005, PMID: 22483681 PMC3568257

[ref13] Dmochowski J. P. Datta A. Bikson M. Su Y. Parra L. C. (2011). Optimized multi-electrode stimulation increases focality and intensity at target. J. Neural Eng. 8:046011. doi: 10.1088/1741-2560/8/4/046011, PMID: 21659696

[ref14] Dundas J. E. Thickbroom G. W. Mastaglia F. L. (2007). Perception of comfort during transcranial DC stimulation: effect of NaCl solution concentration applied to sponge electrodes. Clin. Neurophysiol. 118, 1166–1170. doi: 10.1016/j.clinph.2007.01.010, PMID: 17329167

[ref15] Elias G. J. B. Namasivayam A. A. Lozano A. M. (2018). Deep brain stimulation for stroke: current uses and future directions. Brain Stimul. 11, 3–28. doi: 10.1016/j.brs.2017.10.005, PMID: 29089234

[ref16] Esmaeilpour Z. Kronberg G. Reato D. Parra L. C. Bikson M. (2021). Temporal interference stimulation targets deep brain regions by modulating neural oscillations. Brain Stimul. 14, 55–65. doi: 10.1016/j.brs.2020.11.007, PMID: 33186778 PMC9382891

[ref17] Fernández-Corazza M. Turovets S. Muravchik C. H. (2020). Unification of optimal targeting methods in transcranial electrical stimulation. NeuroImage 209:116403. doi: 10.1016/j.neuroimage.2019.116403, PMID: 31862525 PMC7110419

[ref18] Fischl B. (2012). FreeSurfer. Neuroimage 62, 774–781. doi: 10.1016/j.neuroimage.2012.01.021, PMID: 22248573 PMC3685476

[ref19] Fonov V. Evans A. C. Botteron K. Almli C. R. McKinstry R. C. Collins D. L.. (2011). Unbiased average age-appropriate atlases for pediatric studies. Neuroimage 54, 313–327. doi: 10.1016/j.neuroimage.2010.07.033, PMID: 20656036 PMC2962759

[ref20] Francis J. T. Gluckman B. J. Schiff S. J. (2003). Sensitivity of neurons to weak electric fields. J. Neurosci. 23, 7255–7261. doi: 10.1523/JNEUROSCI.23-19-07255.2003, PMID: 12917358 PMC6740448

[ref21] Fresnoza S. Christova M. Feil T. Gallasch E. Körner C. Zimmer U.. (2018). The effects of transcranial alternating current stimulation (tACS) at individual alpha peak frequency (iAPF) on motor cortex excitability in young and elderly adults. Exp. Brain Res. 236, 2573–2588. doi: 10.1007/s00221-018-5314-3, PMID: 29943239 PMC6153871

[ref22] Gomez-Tames J. Asai A. Hirata A. (2020a). Significant group-level hotspots found in deep brain regions during tDCS: a computational analysis of electric field. Clin. Neurophysiol. 131, 755–765. doi: 10.1016/j.clinph.2019.11.018 31839398

[ref23] Gomez-Tames J. Asai A. Hirata A. (2021). Multiscale computational model reveals nerve response in a mouse model for temporal interference brain stimulation. Front. Neurosci. 15:15. doi: 10.3389/fnins.2021.684465 PMC8277927 34276293

[ref24] Gomez-Tames J. Asai A. Mikkonen M. Laakso I. Tanaka S. Uehara S.. (2019). Group-level and functional-region analysis of electric-field shape during cerebellar transcranial direct current stimulation with different electrode montages. J. Neural Eng. 16:036001. doi: 10.1088/1741-2552/ab0ac5, PMID: 30808008

[ref25] Gomez-Tames J. Hamasaka A. Hirata A. Laakso I. Lu M. Ueno S. (2020b). Group-level analysis of induced electric field in deep brain regions by different TMS coils. Phys. Med. Biol. 65:025007. doi: 10.1088/1361-6560/ab5e4a, PMID: 31796653

[ref26] Gomez-Tames J. Laakso I. Haba Y. Hirata A. Poljak D. Yamazaki K. (2017). Computational artifacts of the in situ electric field in anatomical models exposed to low-frequency magnetic field. IEEE Trans Electromagn Compat. 60, 589–597. doi: 10.1109/TEMC.2017.2748219

[ref27] Gonçalves S. I. De Munck J. C. Verbunt J. P. A. Bijma F. Heethaar R. M. Lopes da Silva F. (2003). In vivo measurement of the brain and skull resistivities using an EIT-based method and realistic models for the head. I.E.E.E. Trans. Biomed. Eng. 50, 754–767. doi: 10.1109/TBME.2003.812164, PMID: 12814242

[ref28] Grossman N. Bono D. Dedic N. Kodandaramaiah S. B. Rudenko A. Suk H. J.. (2017). Noninvasive deep brain stimulation via temporally interfering electric fields. Cell 169, 1029–1041.e16. doi: 10.1016/j.cell.2017.05.024, PMID: 28575667 PMC5520675

[ref29] Gunalan K. Howell B. McIntyre C. C. (2018). Quantifying axonal responses in patient-specific models of subthalamic deep brain stimulation. NeuroImage 172, 263–277. doi: 10.1016/j.neuroimage.2018.01.015, PMID: 29331449 PMC5910209

[ref1002] Hamajima H. Gomez-Tames J. Uehara S. Otaka Y. Tanaka S. Hirata A.. (2023). Computation of group-level electric field in lower limb motor area for different tDCS montages. Clin. Neurophysiol. 150, 69–78. doi: 10.1016/j.clinph.2023.03.009, PMID: 37023635

[ref30] Holtzheimer P. E. Husain M. M. Lisanby S. H. Taylor S. F. Whitworth L. A. McClintock S.. (2017). Subcallosal cingulate deep brain stimulation for treatment-resistant depression: a multisite, randomised, sham-controlled trial. Lancet Psychiatry 4, 839–849. doi: 10.1016/S2215-0366(17)30371-1, PMID: 28988904

[ref31] Huang Y. Liu A. A. Lafon B. Friedman D. Dayan M. Wang X.. (2017). Measurements and models of electric fields in the in vivo human brain during transcranial electric stimulation. Elife 6:e18834. doi: 10.7554/eLife.18834, PMID: 28169833 PMC5370189

[ref32] Huang Y. Parra L. C. (2019). Can transcranial electric stimulation with multiple electrodes reach deep targets? Brain Stimul. 12, 30–40. doi: 10.1016/j.brs.2018.09.010, PMID: 30297323 PMC6301116

[ref33] Hunold A. Haueisen J. Nees F. Moliadze V. (2023). Review of individualized current flow modeling studies for transcranial electrical stimulation. J Neurosci Res 101, 405–423. doi: 10.1002/jnr.25154, PMID: 36537991

[ref34] Indahlastari A. Albizu A. O’Shea A. Forbes M. A. Nissim N. R. Kraft J. N.. (2020). Modeling transcranial electrical stimulation in the aging brain. Brain Stimul. 13, 664–674. doi: 10.1016/j.brs.2020.02.007, PMID: 32289695 PMC7196025

[ref35] Jiang H. Wang M. Wu D. Zhang J. Zhang S. (2022). In vivo measurements of transcranial electrical stimulation in lesioned human brain: a case report. Brain Sci. 12:1455. doi: 10.3390/brainsci12111455 36358381 PMC9688390

[ref36] Kar K. Duijnhouwer J. Krekelberg B. (2017). Transcranial alternating current stimulation attenuates neuronal adaptation. J. Neurosci. 37, 2325–2335. doi: 10.1523/JNEUROSCI.2266-16.2016, PMID: 28137971 PMC5354346

[ref37] Kasten F. H. Duecker K. Maack M. C. Meiser A. Herrmann C. S. (2019). Integrating electric field modeling and neuroimaging to explain inter-individual variability of tACS effects. Nat Commun 10:5427. doi: 10.1038/s41467-019-13417-6, PMID: 31780668 PMC6882891

[ref38] Katoch N. Kim Y. Choi B. K. Ha S. W. Kim T. H. Yoon E. J.. (2023). Estimation of brain tissue response by electrical stimulation in a subject-specific model implemented by conductivity tensor imaging. Front. Neurosci. 17:17. doi: 10.3389/fnins.2023.1197452 PMC10242016 37287801

[ref39] Khan A. Yuan K. Bao S. C. Ti C. H. E. Tariq A. Anjum N.. (2022). Can transcranial electrical stimulation facilitate post-stroke cognitive rehabilitation? A systematic review and Meta-analysis. Front. Rehabilit. Sci. 3:3. doi: 10.3389/fresc.2022.795737 PMC9397778 36188889

[ref40] Laakso I. Mikkonen M. Koyama S. Hirata A. Tanaka S. (2019). Can electric fields explain inter-individual variability in transcranial direct current stimulation of the motor cortex? Sci Rep. 9:626. doi: 10.1038/s41598-018-37226-x 30679770 PMC6345748

[ref41] Laakso I. Tanaka S. Mikkonen M. Koyama S. Sadato N. Hirata A. (2016). Electric fields of motor and frontal tDCS in a standard brain space: a computer simulation study. Neuroimage 137, 140–151. doi: 10.1016/j.neuroimage.2016.05.032, PMID: 27188218

[ref42] Lee S. Lee C. Park J. Im C. H. (2020). Individually customized transcranial temporal interference stimulation for focused modulation of deep brain structures: a simulation study with different head models. Sci. Rep. 10, 1–11. doi: 10.1038/s41598-020-68660-5 32678264 PMC7366675

[ref43] Li L. M. Violante I. R. Leech R. Ross E. Hampshire A. Opitz A.. (2019). Brain state and polarity dependent modulation of brain networks by transcranial direct current stimulation. Hum. Brain Mapp. 40, 904–915. doi: 10.1002/hbm.24420, PMID: 30378206 PMC6387619

[ref44] Liew S. L. Anglin J. M. Banks N. W. Sondag M. Ito K. L. Kim H.. (2018). A large, open source dataset of stroke anatomical brain images and manual lesion segmentations. Sci Data. 5:5. doi: 10.1038/sdata.2018.11 PMC5819480 29461514

[ref45] McCann H. Beltrachini L. (2021). Does participant’s age impact on tDCS induced fields? Insights from computational simulations. Biomed Phys Eng Express. 7:045018. doi: 10.1088/2057-1976/ac0547, PMID: 34038881

[ref46] Middleton F. A. Strick P. L. (2000). Basal ganglia and cerebellar loops: motor and cognitive circuits. Brain Res. Rev. 31, 236–250. doi: 10.1016/S0165-0173(99)00040-5, PMID: 10719151

[ref47] Minjoli S. Saturnino G. B. Blicher J. U. Stagg C. J. Siebner H. R. Antunes A.. (2017). The impact of large structural brain changes in chronic stroke patients on the electric field caused by transcranial brain stimulation. Neuroimage Clin. 15, 106–117. doi: 10.1016/j.nicl.2017.04.014 28516033 PMC5426045

[ref48] Mirzakhalili E. Barra B. Capogrosso M. Lempka S. F. (2020). Biophysics of temporal interference stimulation. Cell Syst. 11, 557–572.e5. doi: 10.1016/j.cels.2020.10.004, PMID: 33157010

[ref49] Mizutani-Tiebel Y. Takahashi S. Karali T. Mezger E. Bulubas L. Papazova I.. (2022). Differences in electric field strength between clinical and non-clinical populations induced by prefrontal tDCS: a cross-diagnostic, individual MRI-based modeling study. Neuroimage Clin. 34:103011. doi: 10.1016/j.nicl.2022.103011 35487132 PMC9125784

[ref50] Naros G. Gharabaghi A. (2017). Physiological and behavioral effects of β-tACS on brain self-regulation in chronic stroke. Brain Stimul. 10, 251–259. doi: 10.1016/j.brs.2016.11.003, PMID: 27965067

[ref51] Opitz A. Yeagle E. Thielscher A. Schroeder C. Mehta A. D. Milham M. P. (2018). On the importance of precise electrode placement for targeted transcranial electric stimulation. NeuroImage 181, 560–567. doi: 10.1016/j.neuroimage.2018.07.027, PMID: 30010008 PMC6139038

[ref52] Ozen S. Sirota A. Belluscio M. A. Anastassiou C. A. Stark E. Koch C.. (2010). Transcranial electric stimulation entrains cortical neuronal populations in rats. J Neurosci 30, 11476–11485. doi: 10.1523/JNEUROSCI.5252-09.2010, PMID: 20739569 PMC2937280

[ref53] Pauli W. M. Nili A. N. Michael T. J. (2018). Data descriptor: a high-resolution probabilistic in vivo atlas of human subcortical brain nuclei. Sci Data. 5:5. doi: 10.1038/sdata.2018.63 PMC5903366 29664465

[ref54] Plonsey R. Heppner D. B. (1967). Considerations of quasi-stationarity in electrophysiological systems. Bull Math Biophys 29, 657–664. doi: 10.1007/BF02476917, PMID: 5582145

[ref55] Puonti O. Saturnino G. B. Madsen K. H. Thielscher A. (2020). Value and limitations of intracranial recordings for validating electric field modeling for transcranial brain stimulation. NeuroImage 208:116431. doi: 10.1016/j.neuroimage.2019.116431, PMID: 31816421

[ref56] Puonti O. Van Leemput K. Saturnino G. B. Siebner H. R. Madsen K. H. Thielscher A. (2020). Accurate and robust whole-head segmentation from magnetic resonance images for individualized head modeling. NeuroImage 219:117044. doi: 10.1016/j.neuroimage.2020.117044, PMID: 32534963 PMC8048089

[ref57] Rampersad S. Roig-Solvas B. Yarossi M. Kulkarni P. P. Santarnecchi E. Dorval A. D.. (2019). Prospects for transcranial temporal interference stimulation in humans: a computational study. NeuroImage 202:116124. doi: 10.1016/j.neuroimage.2019.116124 31473351 PMC6819277

[ref58] Rezaee Z. Dutta A. (2019). Cerebellar lobules optimal stimulation (CLOS): a computational pipeline to optimize cerebellar lobule-specific electric field distribution. Front. Neurosci. 13:266. doi: 10.3389/fnins.2019.00266 31031578 PMC6473058

[ref59] Richter L. Neumann G. Oung S. Schweikard A. Trillenberg P. (2013). Optimal coil orientation for transcranial magnetic stimulation. PLoS One 8:e60358. doi: 10.1371/journal.pone.0060358, PMID: 23593200 PMC3623976

[ref60] Saturnino G. B. Antunes A. Thielscher A. (2015). On the importance of electrode parameters for shaping electric field patterns generated by tDCS. NeuroImage 120, 25–35. doi: 10.1016/j.neuroimage.2015.06.067, PMID: 26142274

[ref61] Soldati M. Laakso I. (2020). Computational errors of the induced electric field in voxelized and tetrahedral anatomical head models exposed to spatially uniform and localized magnetic fields. Phys Med Biol 65:015001. doi: 10.1088/1361-6560/ab5dfb 31791030

[ref62] Soleimani G. Saviz M. Bikson M. Towhidkhah F. Kuplicki R. Paulus M. P.. (2021). Group and individual level variations between symmetric and asymmetric DLPFC montages for tDCS over large scale brain network nodes. Sci Rep 11, 1–13. doi: 10.1038/s41598-020-80279-0 33446802 PMC7809198

[ref63] Song X. Zhao X. Li X. Liu S. Ming D. (2020). Multi-channel transcranial temporally interfering stimulation (tTIS): application to living mice brain. J. Neural Eng. 18:036003. doi: 10.1088/1741-2552/abd2c9 33307539

[ref64] Stoupis D. Samaras T. (2022). Non-invasive stimulation with temporal interference: optimization of the electric field deep in the brain with the use of a genetic algorithm. J. Neural Eng. 19:056018. doi: 10.1088/1741-2552/ac89b3, PMID: 35970146

[ref65] Thielscher A. Antunes A. Saturnino G. B. Field Modeling for Transcranial Magnetic Stimulation: A Useful Tool to Understand the Physiological Effects of TMS? In: Proceedings of the Annual International Conference of the IEEE Engineering in Medicine and Biology Society, EMBS; (2015). 10.1109/EMBC.2015.7318340 26736240

[ref66] Violante I. Alania K. Cassarà A. Neufeld E. Acerbo E. Williamson A.. (2023). Non-invasive temporal interference electrical stimulation of the human hippocampus. Brain Stimul. 16:408. doi: 10.1016/j.brs.2023.01.833 PMC10620081 37857775

[ref67] Volkow N. D. Wise R. A. Baler R. (2017). The dopamine motive system: implications for drug and food addiction. Nat. Rev. Neurosci. 18, 741–752. doi: 10.1038/nrn.2017.130, PMID: 29142296

[ref68] von Conta J. Kasten F. H. Ćurčić-Blake B. Aleman A. Thielscher A. Herrmann C. S. (2021). Interindividual variability of electric fields during transcranial temporal interference stimulation (tTIS). Sci. Rep. 11:20357. doi: 10.1038/s41598-021-99749-0 34645895 PMC8514596

[ref69] Wagner T. A. Zahn M. Grodzinsky A. J. Pascual-Leone A. (2004). Three-dimensional head model simulation of transcranial magnetic stimulation. IEEE Trans Biomed Eng 51, 1586–1598. doi: 10.1109/TBME.2004.827925 15376507

[ref70] Wang M. Feng T. Jiang H. Zhu J. Feng W. Chhatbar P. Y.. (2022). In vivo measurements of electric fields during cranial electrical stimulation in the human brain. Front. Hum. Neurosci. 16:829745. doi: 10.3389/fnhum.2022.829745 35250520 PMC8895368

[ref71] Wang M. Lou K. Liu Z. Wei P. Liu Q. (2023). Multi-objective optimization via evolutionary algorithm (MOVEA) for high-definition transcranial electrical stimulation of the human brain. Neuroimage 280:120331. doi: 10.1016/j.neuroimage.2023.120331 37604295

[ref72] Zhu X. Li Y. Zheng L. Shao B. Liu X. Li C.. (2019). Multi-point temporal interference stimulation by using each electrode to carry different frequency currents. IEEE Access. 7, 168839–168848. doi: 10.1109/ACCESS.2019.2947857

